# A qualitative study of police officers’ experiences of solidarity and belonging through social activities

**DOI:** 10.1371/journal.pone.0360545

**Published:** 2026-11-12

**Authors:** Patrik Thunholm, Ingeborg Nilsson, Elin Granholm Valmari

**Affiliations:** 1 Communications Department, Swedish Police Authority, Stockholm, Sweden; 2 Department of Community Medicine and Rehabilitation, Umeå University, Umeå, Sweden; Instituto Federal Goiano, BRAZIL

## Abstract

**Introduction:**

Research on social participation has primarily focused on community and ageing populations, leaving a gap in understanding how social participation is experienced within other contexts, such as high-stress professions, where group cohesion is vital to health. This study aims to explore continuities and changes in social participation among Swedish police officers over time and to examine how these patterns relate to contemporary officers’ experiences of social participation and solidarity.

**Method:**

We used a historiographical method, analysing document data and interviews through qualitative content analysis. A compilation of 120 years of data from police union journals, along with 17 semi-structured interviews with officers, was utilised.

**Results:**

Shared social activities were described as fostering belonging and solidarity among police officers. The findings suggest that social participation may support cohesion in high-stress professions, while solidarity may also be associated with exclusionary norms related to gender and emotional openness.

**Conclusion:**

Findings extend current conceptualisations of social participation by illustrating how social activities in the police profession may be linked to solidarity. The study also highlights how occupational cultures shape the meanings and health implications of social participation over time, offering insights relevant to other high-stress professions.

## Introduction

Social participation, a subjective experience of meaningful engagement in social activities [1], plays a key role in shaping people’s lifestyles. It is influenced by interactions across diverse social contexts and environments [2] and changes in response to available time and resources [1]. A taxonomy of social participation was provided by Levasseur et al. [3]. The taxonomy guides research and practice, and distinguishes social engagement from activity goals across six levels: ‘Doing an activity in preparation for connecting with others’, ‘Being with others’, ‘Interacting with others without doing a specific activity with them’, ‘Doing an activity with others’, ‘Helping others’, and ‘Contributing to society’ [3]. Although Levasseur et al.’s [3] taxonomy provides a valuable framework for classifying levels of social participation, it was developed primarily within ageing and public health contexts. Consequently, its applicability to coercive state professions characterised by strong internal solidarity, organisational stressors, and collective coping practices remains insufficiently explored. Within such occupational cultures, social participation may extend beyond activity engagement to foster belonging, cohesion, and occupational resilience. However, empirical knowledge of how these dynamics are expressed in everyday professional life, and how they have evolved over time, remains limited. Hence, given that social participation supports health [4], examining how it is experienced in demanding occupational settings is relevant, especially as trust and a sense of belonging are central to well-being [5,6].

Different aspects of social participation are widely recognised as health determinants [4], reducing loneliness and serving as a buffer against socioeconomic disadvantages [7]. People with strong social connections live longer and experience better physical and mental health [8]. Moreover, organisational social support is particularly beneficial in high-demanding contexts [9], alleviating the severity of trauma [10].

A key aspect of social engagement is belonging, the subjective sense of being accepted, valued, and connected through shared activity experiences, alongside the group cohesion that arises from social interaction [11]. Social support and group cohesion are vital for fostering workplace cohesion and solidarity. Solidarity denotes social integration, contrasting with coercion or self-interest. Solidarity encompasses group membership, from friendships to national camaraderie, and fosters group cohesion, mutual support, and cultural norms [12]. High-demand professions, such as the police profession, accentuate this dynamic because they hold state-sanctioned coercive authority while remaining under democratic scrutiny. The resulting ‘outsider-insider’ tension pushes the profession to cultivate strong inward solidarity [13], which is intertwined with resilience and health, as officers rely on their professional networks to manage work demands [14]. For instance, prior research has shown that forming tight-knit groups helps alleviate the psychological burden of work tasks. Social support has also been found to protect against ill-health following potentially traumatic events [15].

Social participation among officers has historically evolved from local, community-based activities to more globalised and digital forms of interaction [16]. In the early 20th century, police social involvement included church attendance and local events, with parks and community halls as key hubs for interaction [17]. The introduction of radio expanded access to news and entertainment within the home, contributing to changes in how leisure time was spent and potentially reducing reliance on public settings for shared activities [18]. Legislation on working hours and holidays improved working conditions, while unions and social movements strengthened the rights of officers [19]. After World War II, interest in social clubs grew, while television further contributed to a shift towards home-based leisure and shared family activities, changing the social contexts in which leisure and interaction took place [20]. The technological revolution, including social media and constant connectivity, radically transformed officers’ social interactions [21].

In summary, research examining how social participation is historically constituted within occupational cultures remains scarce. Limited attention has also been given to how social participation is structured, experienced, and sustained within high-demand, male-dominated professions such as policing, where occupational culture, hierarchy, and exposure to trauma shape social interaction differently. A historical perspective may be particularly valuable in this regard [22], as it can identify continuities and changes in how social participation has been represented within policing over time. However, documentary sources alone cannot capture how police officers experience social participation today. Combining longitudinal documentary material with contemporary interviews therefore enables examination of both temporal patterns and their relation to current experiences of social participation and solidarity. This study therefore aims to explore continuities and changes in social participation among Swedish police officers over time and to examine how these patterns relate to contemporary officers’ experiences of social participation and solidarity.

## Method

### Research design

Historical methods, underutilised in health science, help trace activities and contexts over time, revealing cultural, social, and institutional influences on engagement. This study used a historiographical approach [22] comprising nine steps and combining document data with interviews: (1) formulate a research question, (2) check relevance, (3) define scope, (4) gather sources, (5) assess analysis methods, (6) assemble evidence, (7) develop the story, (8) critique it, and (9) define outcomes [22]. Steps 7–8 are presented in the results, and steps 8–9 are presented in the discussion. We have followed the historiographical methodology in every stage, as described by O’Brien, Remenyi, and Keaney [22]. The two data sources served complementary analytical purposes. The longitudinal documentary material enabled the examination of continuities and changes in how social participation was represented over time, while the interviews provided first-person accounts of how social participation was experienced by contemporary police officers. Integrating the two sources enabled the longitudinal patterns identified in the documentary material to be examined in relation to contemporary experiences. Ethical approval for the interviews was obtained from the Swedish Ethical Review Authority on 25 January 2021 (Reg No. 2020–07170), in accordance with the Helsinki Declaration [23]. Participants signed informed consent to participate and were informed, orally and in writing, of their right to withdraw at any stage without providing a reason. We removed identifying details from transcripts, and anonymised quotations to prevent recognition of individuals or units. All data were accessible only to the research team, in accordance with the university’s data protection regulations. As interviews touched on occupational stress, trauma exposure, and workplace dynamics, participants were reminded that they could refrain from answering any question that caused discomfort.

In terms of reflexivity, the research team’s disciplinary backgrounds in occupational science, social participation, and historical policing research informed the analytical lens used to analyse the material. Reflexive discussions between authors were held throughout the analysis to critically explore how prior knowledge and theoretical backgrounds may have shaped the interpretation of the data.

The study was reported in accordance with established qualitative research reporting standards, specifically the Journal Article Reporting Standards for Qualitative Research (JARS-Qual) [24], to enhance methodological transparency and rigor.

### Determine the scope and obtain sources of data

A 120-year period was considered sufficient for the study. A table of contents from Swedish police trade union journals (1904–2023) was compiled, and relevant articles were identified, i.e., all articles focusing on social activities in line with Levasseur’s taxonomy. The journals are directed primarily towards members of the Swedish Police Union and cover issues considered current and relevant to police officers, including working conditions, organisational and professional issues, trade union matters, and topics of broader interest to the profession. Journalists mainly write the articles, but the journals also include contributions from police officers, such as letters and opinion pieces. Thus, the document material provides a member-oriented perspective on issues and experiences considered relevant to police officers and their professional and social lives. The interview material complemented this documentary perspective by providing first-person accounts of contemporary officers’ experiences of social participation in everyday life. The journal material was treated as a longitudinal documentary source spanning 1904–2023 rather than as a discrete historical period contrasted with the interviews. Accordingly, we applied no temporal cut-off between historical and contemporary material; the later journal articles intentionally overlap with the interviews conducted in 2021. A supplementary file lists them by decade, title, and summary (See S1 File).

The interview data were gathered between 15/02/2021and 31/08/2021 through semi-structured interviews with 17 Swedish officers (aged 29–51, with an average of 8.7 years of work experience), including six women. Convenience sampling was initially used, followed by snowball and purposive sampling to maximise variation. Hence, participants were recruited to achieve experiential variation in age, gender, workplace, and years of service. No exclusion criteria were applied. All participants were frontline officers to provide a consistent contemporary occupational perspective on social participation within the population of interest. Recruitment followed the principle of information power, ending when sufficient data had been gathered to address the study’s aim [25].

JARS-Qual reporting guideline was utilised to plan and conduct the interviews [24]. First, a semi-structured interview guide was developed focusing on officers’ lifestyles. The semi-structured interview guide included open-ended main questions designed to elicit participants’ experiences of social participation within and beyond the workplace. For instance, what types of social activities police officers engage in on a daily basis; how they experience communication; what types of social relationships they have in life and how they experience these relationships; what types of social roles they have besides their police role and how they experience these roles. Furthermore, the type of support they receive in everyday life. All questions were asked about both work and private life, and follow-up probes were used to deepen reflection and elicit concrete examples.

The interview guide was pilot tested prior to the interviews. Interviews were conducted by the last author and lasted approximately two hours, taking place at locations chosen by the participants for their convenience, such as their workplaces, private meeting rooms, or secure videoconferencing platforms, depending on their preferences and geographical locations. Efforts were made to ensure a confidential setting where participants could speak freely without interruption. All recordings were transcribed verbatim by a medical secretary. Transcripts were reviewed against audio files to ensure accuracy, and identifying details were removed during transcription to protect participant confidentiality.

### Assess the methods of analysis and assemble the evidence

Data analysis proceeded in several steps. First, the data were repeatedly read to identify meaning units related to social participation and to achieve familiarity with the material. All materials, both interviews and document data, were read repeatedly before coding. This iterative reading process supported experiential accounts across both datasets.

Second, condensation and deductive coding were conducted in line with Elo and Kyngäs [26], which guided the initial coding process. For instance, meaning units relating to social participation were identified by one author. Another author then conducted deductive coding based on Levasseur et al.’s [3] final four levels in the taxonomy: “Interacting with others,” “Doing an activity with others,” “Helping others,” and “Contributing to society”, as we focused on actual social activities and the taxonomy distinguishes forms of social participation by activity type. Coding was conducted in MAXQDA [27].

Third, we examined the preliminary categories within each data source and then across the document and interview material. The two sources were subsequently integrated in an iterative analytical process to identify continuities, changes, and recurring patterns in social participation over time, as well as how these patterns related to contemporary officers’ experiences. We first clustered the data by social activity type, such as musical activities, playing chess, excursions, parties, and sobriety activities. These clusters formed preliminary categories, which we examined across time periods and data sources to support developing the final themes. As the material extended beyond Levasseur et al.’s taxonomy [3], particularly around solidarity, we used an inductive, collaborative approach to generate themes. We documented analytic decisions and theme development throughout to establish an audit trail. We triangulated findings through iterative comparison of patterns and interpretations across document and interview materials, along with discussions within the research team to challenge and refine emerging interpretations until we reached consensus.

Lastly, we created a timeline of selected events to illustrate social activities over time, and used quotations from the semi-structured interviews to show how contemporary officers experienced and understood the identified patterns [22]. The timeline presents selected illustrative examples chosen to represent variation across decades rather than exhaustive coverage of all identified materials.

## Results

Findings revealed that social and recreational activities described across the document and interview material, ranging from early 20th-century sports contests to modern-day wellness initiatives, functioned as meaningful forms of shared social participation. Fig 1 provides a visual summary of examples, and the supplementary file (S1 File) includes a summary of all document data. The timeline presents selected illustrative markers. Therefore, periods with fewer markers, e.g., 1946–1960, do not indicate an absence of specific social activities or related topics.

**Fig 1:**
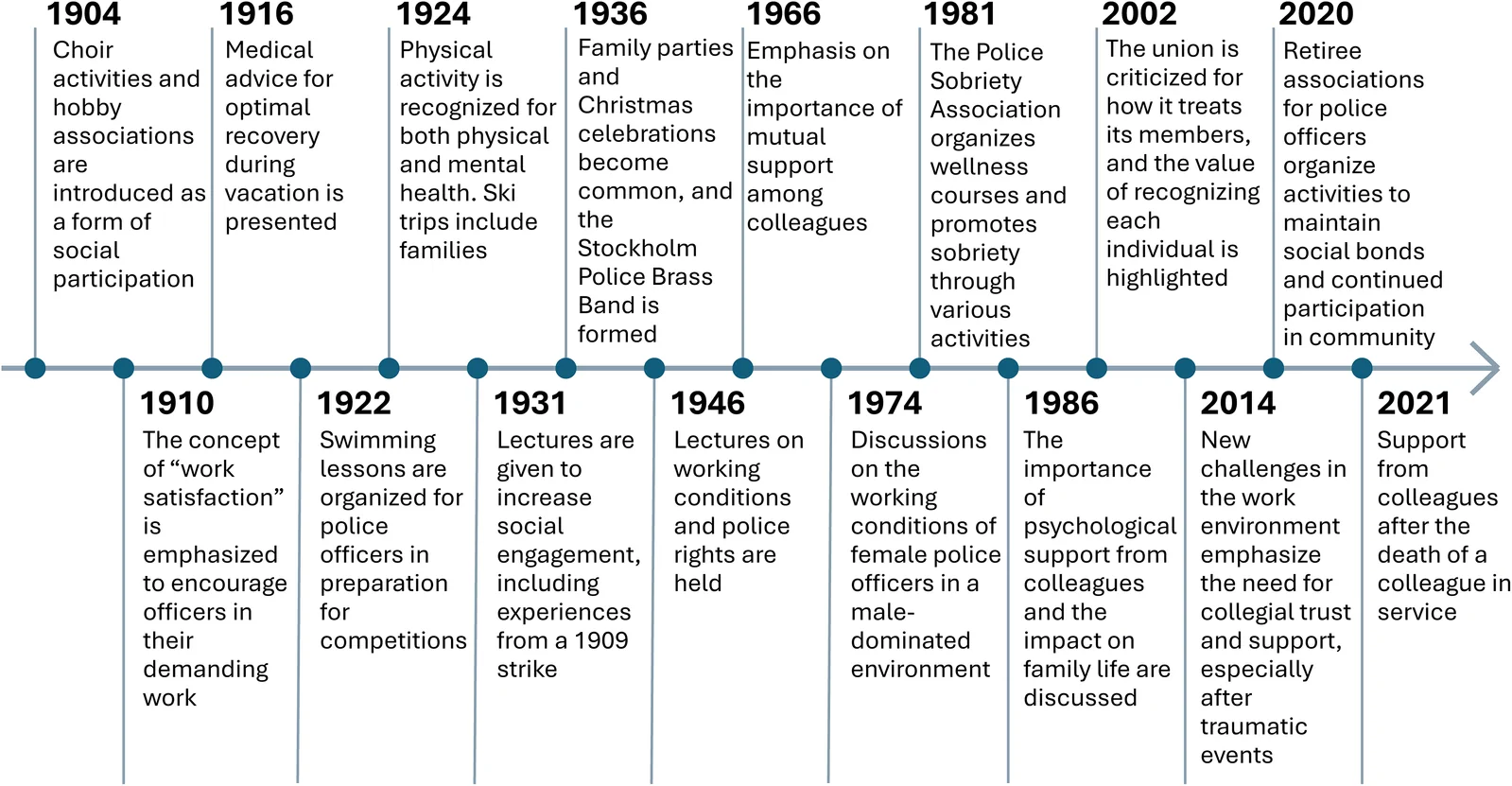
An Overview of Examples Presented on a Historical Timeline. The figure represents examples for each decade. See the supplementary file for all historical data included.

The data revealed two main themes: “Encouraging Social Engagement Leading to Broader Social Participation” and “Fostering Camaraderie, Including Solidarity and a Deep Sense of Belonging.” The two themes describe recurring patterns in how social activities were represented and experienced across the document and interview material, particularly in relation to social engagement, camaraderie, solidarity, and belonging. This solidarity may serve as a health-promoting resource, extending existing taxonomies by capturing how belonging is integrated with social participation in high-demand contexts. See Table 1 for a table of themes.

**Table 1:** Overview of the different themes in the study.

*Encouraging Social Engagement Leading to Broader Social Participation*	*Fostering Camaraderie, Including Solidarity and a Deep Sense of Belonging*
This theme explores how police officers encourage social engagement and social participation through various social activities across different levels. Initiatives ranged from establishing health and wellness programmes (preparing for interaction) to participating in sports and competitions (interacting with others), involving community and family in events and other recreational activities (engaging in social activities), engaging in international collaborations (contributing to society), and maintaining social connectedness post-retirement (continuity in social participation). These forms of social engagement were associated with social connection, mutual support, and continued social participation across officers’ careers and beyond. In summary, the interviews illustrated how these forms of social participation continue today, particularly through informal physical activities, peer interaction during shifts, and individually initiated social engagement negotiated alongside family life and recovery needs.	This theme examines how police officers fostered camaraderie, solidarity, and a deep sense of belonging within the force, aligning with both macro- and micro-level behaviours and attitudes that explain solidarity. Key aspects include promoting unity and social support to strengthen group cohesion, advancing social education and inclusivity to enhance solidarity, adapting to increasing diversity to promote social integration, addressing gender dynamics to improve social interactions and sense of belonging, and supporting health and well-being during crises to reinforce solidarity and social support. Across the material, camaraderie, solidarity, and belonging were associated with group cohesion, mutual support, and meaningful social connections within the occupational group. In summary, the interviews further highlighted how camaraderie is experienced through emotional support after critical incidents, everyday peer dialogue, shared humour, and a sense of being understood within the occupational group.

### Encouraging social engagement leading to broader social participation

This theme, spanning 1904–2023, describes recurring forms of social engagement represented across the document and interview material, including health initiatives, sports, community events, and cross-professional collaborations. Across the material, these activities were associated with social interaction, mutual support, and participation within and beyond the occupational group.

#### Early representation of social engagement through health and wellness initiatives.

Health-related forms of social engagement were represented early in the document material. A 1910 article described”work happiness”, emphasising the need for encouragement due to the thankless nature of police work. In 1916, a doctor offered advice on how to achieve optimal recovery during vacation. By 1931, lectures were being given on various topics to increase social engagement. For instance, a 1931 lecture discussed the experiences of a 1909 strike, and a 1946 lecture covered working conditions. In 1979, a research report highlighted physical, psychological, and social difficulties associated with working irregular hours.

Discussions about vacations occurred early, with a 1917 proposal for a police recreation home reflecting the demands of the profession. In 1947, social interaction with others for personal development was highlighted. Furthermore, diverse study circles were established to promote social engagement in various aspects of health. Between the 1950s and 1980s, various events, including philosophical discussions, dance classes, skipper’s certificate courses, and study trips, were organised. Alcohol-related discussions appeared recurrently across the archival material, often in connection with sobriety associations, educational initiatives, and occupational health concerns. These discussions focused on the social and professional risks of alcohol use, as well as prevention and support initiatives within the police community. For instance, the Swedish Police Sobriety Association organised wellness courses in 1981, promoting health and sobriety through social engagement in different conferences and courses. By the 1980s, officers were advocating for wellness weeks to address work-related health issues, and the police organisation mandated health checks focusing on stress and psychosocial work environment challenges. The interview data also highlighted contemporary concerns about officers’ physical health and access to preventive support, as illustrated by this female officer: *“…people are having problems with their backs and legs…we really need more help, like from physiotherapists and such…It’s almost a miracle that more people aren’t on sick leave because of pain.”*

#### Participating in physical activities, sports, and competitions.

Physical activities, sports, and competitions were represented early in the document material as recurring forms of social engagement among officers. In 1922, swimming lessons were organised for officers in preparation for competitions. By 1924, physical activity was recognised not only for its health benefits but also for its contribution to mental well-being. Social engagement through sports, physical activities, and competitions was encouraged to foster social interaction and participation. Various physical activities, including skiing, orienteering, wrestling, and fencing, were organised, with different championships held between 1947 and 1987. Weightlifting events were documented as early as 1946, while discussions about handball and shooting had increased by 1947. Orienteering competitions are described in the data up to 2022, while shooting events and pistol tournaments are described up to 2021. Dog-handling competitions were first reported in the material in 1989.

The interview data also described physical activity as a contemporary context for social interaction among officers, although participation was sometimes negotiated alongside family life and other everyday demands. Officers organised and engaged in physical activities together, both at work and outside of work; they also work out individually alongside others. One male officer describes the different possibilities for socialising while engaging in physical activities*; “It’s not like we have any organised training... People just do...different things, usually based on personal interests. There’s a large sports hall, a gym, and a training hall...”*

#### Community, family, and recreational activities.

Family- and community-oriented events were recurrently represented as forms of social engagement in the document material. First described in 1936, family parties, including annual Christmas celebrations, were common. Families were invited to participate in social events and gatherings, e.g., a skiing outing in 1924 and a study circle in 1931, which featured music and a speaker from Antwerp who was a former sailor. There were also family-inclusive work and leisure trips, such as a trip to Paris in 1925. Similar trips continued into the 1940s and 1950s.

A Swedish police church service was reported in 1946, followed by Christian community conferences during the 1980s. Choirs were formed as early as 1904, with the Stockholm Police Brass Band being described in 1947. Hobby associations and music events were common during the 1940s. Finally, social activities such as chess competitions were reported as early as 1915, and chess and bridge competitions were described until the 1980s. In 1988, “The Police Day” was organised in a Swedish town as a public event, featuring demonstrations that showcased police skills and fostered respect and understanding between the police and the community. Compared with the family- and community-inclusive activities represented in the earlier journal material, the interview accounts more commonly described informal social participation centred on close colleagues. The interviews included accounts of officers spending time with colleagues outside work through different recreational activities, as illustrated by this female officer: *“We usually try to come up with different activities…Most of my friends are from work.”*

#### International and cross-professional collaboration.

Engaging socially in ways that extend beyond the police profession and national borders has been on the agenda for officers. In 1933, a choir performance was held in collaboration with the fire brigade. In 1946, an orienteering competition involving officers, fire brigade staff, customs officials, and tramway personnel included shooting and hand grenade throwing. International collaboration gained prominence with the Danish police choir’s visit to Stockholm in 1936. Scandinavian sports competitions took place in 1924, and an article from 1988 mentioned a 1951 study trip to Germany, which led to lasting friendships between officers from different nations. International or cross-professional collaboration did not emerge in the interview material. This difference should be interpreted cautiously, as the absence of such accounts in the interviews does not indicate that these activities no longer occur or have become less important.

#### Fostering camaraderie, including solidarity and a deep sense of belonging.

This theme, based on material spanning 1907–2023, describes recurring representations and experiences of camaraderie, solidarity, and belonging. It includes accounts related to unity and mutual support, social education, gender dynamics, well-being during crises, and belonging beyond active service. Across the material, these aspects were associated with group cohesion and meaningful social connections within the police occupational context. Moreover, they uphold the values essential to their profession, aligning with the micro-level behaviours and attitudes that explain group cohesion, as this male officer:

…being in the police force, there’s a strong sense of camaraderie…many like-minded people…with similar interests, around the same age, and in similar life situations…that, and the variety at work, are the main reasons…why I’m staying...

#### Promoting unity and support for each other.

References to camaraderie and mutual support were present in the document material as early as 1907, when an article emphasised the recruitment of capable and suitable officers. Camaraderie and solidarity were encouraged by promoting unity and mutual care. For example, in 1918, a lottery was approved to finance a recreation home for officers, fostering camaraderie outside of work. Social gatherings also began at the police academy, where teachers and students participated in trips to build cohesion and provide support, as seen in the 1940s. These accounts indicate that solidarity was expressed not only in the workplace but also in personal and recreational contexts.

The risks of internal isolation, which could fragment unity, were highlighted as early as 1919 with discussions on the historical corps spirit. By 1924, criticisms of poor comradeship emerged, calling for improvements in unity. Throughout the 1920s and into the 1930s, articles emphasised the importance of brotherhood, mutual support, and camaraderie. Calls for collective unity and warnings against division appeared in texts from 1933 and 1934, reinforcing the importance of esprit de corps for professional effectiveness. Unification remained a key theme in 1938, with efforts to consolidate fragmented police unions.

Even in the 1960s and 2000s, solidarity was of critical value. In 1966, the importance of mutual support was emphasised. In 2002, criticisms emerged about how the union treated its members, suggesting they should be valued individuals and not taken for granted. The interview material similarly contained accounts of both strong collegial unity and situations in which this unity was challenged. Even if most officers perceive that there is usually a mutual understanding and collaboration among colleagues, there are occasionally incidents that appear to divide the collective unity that has been established. Such an example is provided by one of the male officers; *“…we have this close collaboration… However, over a period... an individual…misused that strength in a way that created intense ripples throughout the entire group, just by the way he acted.”*

#### Social education and inclusivity.

From the 1910s onward, social education was recurrently represented in the document material, with officers encouraged to educate one another on professional, social, and ethical issues. These activities were often discussed in relation to camaraderie, solidarity, and professional standards. Ongoing learning, both during and outside of work hours, was emphasised to foster strong professional and ethical standards in line with policing values. In 1987, a police research symposium was organised. Fostering camaraderie involved guiding one another in moral and professional conduct, e.g., articles throughout the 1930s, 1950s, and 1980s explored the impact of alcohol on social conditions and sobriety as a crucial value. Even in 2006, an article addressed how best to support a colleague struggling with alcohol problems.

As the police force became more diverse, topics related to diversity became more visible. E.g., educational articles from the 1980s to the 2000s challenged stereotypes within the police force, exploring what it meant to be a homosexual man in the police force, how Jehovah’s Witnesses could serve as officers just as effectively as others, or addressed the challenges female officers face nationally and internationally. Topics related to racism became more visible in the document material during the 2000s, alongside broader discussions of diversity, inclusion, and challenges to solidarity within the police force. In 2005, gay officers participated in a pride parade, promoting future inclusivity. However, while perspectives on inclusion have progressed, mental health remains stigmatised, as illustrated by this male officer’s account:

…if you compare it to…how it used to be…there’s more awareness…and…no longer seen as something only for people “with a screw loose”…But I still don’t think it’s completely okay to talk about. Then, there’s the question of what the consequences would be if one did...I’m not really sure why I wouldn’t want to talk about it…I guess it’s about maintaining an image…that you can tough it out.

#### Supporting health and well-being through crisis and trauma.

Accounts of officers supporting one another in relation to health and well-being appeared across the material. These included descriptions of emotional and practical support in response to the physical and psychological demands of police work. Reflections on the meaning of Christmas in 1936 emphasised the importance of mental well-being through stillness and self-reflection. Articles from 1986 described psychological support among police colleagues and also reported that spouses could feel excluded from work-related discussions. Family relations were also addressed in a 1974 article series discussing how police work affected both officers and their families, and in a 1986 survey examining the effects of police work on family life.

Solidarity during traumatic events is another critical aspect. In 1994, articles described emotional support provided after officers neutralised a dangerous murderer, or officers affected, following the *Estonia* ferry disaster on the Baltic Sea. Similarly, a 2019 article described how a stroke-affected officer received support from colleagues and a superior. In 2021, another example showed officers supporting each other after a colleague was fatally shot. Mutual support also emerged in the interviews, as illustrated by this female officer’s account: *“...with the colleagues...it’s extremely important to be able to talk…and I really feel that this atmosphere exists at work.”*

#### Advancing gender dynamics and social interactions.

Gender-related issues became increasingly visible in the document material over time, including discussions of workplace relationships, gender equality, discrimination, bullying, and sexual harassment. For instance, romantic relationships at work were first reported in 2006, with an analysis of the pros and cons of colleagues working and living together. By 2023, a romantic relationship between colleagues was described as potentially leading to misconduct and employer investigations. However, discussions on gender and marital status began as early as 1926, with a city council report on inspections of unmarried police constables’ dormitories, which was considered amusing at the time.

More explicit discussions of gender equality and women’s working conditions appeared in the document material over time. In 1974, a female officer submitted a discussion piece about the working conditions for women in a male-dominated workplace. A 1990s article highlighted female officers’ challenges in securing promotions compared to their male counterparts. Gender discrimination, bullying, and sexual harassment were reported from the 1990s to 2012, highlighting the unique challenges faced by female officers.

Between the 1990s and 2014, group cohesion was discussed in relation to bullying, insufficient psychosocial care after critical incidents, and efforts to improve support and communication among colleagues. There were also articles on how the police authority worked with group cohesion. Both men and women mentioned bullying in the interview data. Among the six female participants in this study, experiences of gender dynamics were mixed, but more often described as workable or improving within their closest teams. However, this should be interpreted cautiously, given the limited number of women included in the interview data. One described the routines established when she joined her group as the only woman; *“…it was very much a ‘guy’ atmosphere. However, …we established boundaries in how we talked to each other... Since then, it has worked well...”*

#### Retirement and belonging beyond work life.

Post-retirement social participation was represented in the document material from the 1970s onward, including study circles, pensioners’ associations, reunions, walks, study visits, and other organised activities. These associations organised various activities, including meetings, reunions, walks, study visits, museum visits, and annual gatherings. In 1989, Stockholm police pensioners established a support fund for retired officers in financial difficulty, demonstrating long-term care for colleagues. A 2020 article described officers who had returned to the profession after leaving, with camaraderie identified as an important reason for returning.

Even after retirement, officers remained engaged in societal roles. A 2014 thesis found many took on responsibilities like managing housing associations or leading community clubs, extending social connectedness beyond the profession. Although the interviews did not address retirement, they included accounts showing that occupational relationships and engagement could extend beyond formal working hours. One male officer says*, “I do live for the job...I always carry my work phone, and I get tips...Nobody wants to sit on hold with 911, and by the time those tips reach us…they’re useless anyway…*

## Discussion

This study explored continuities and changes in social participation among Swedish police officers over time and how these patterns relate to contemporary officers’ experiences. The findings highlight recurring representations and experiences of leisure, sports, family-oriented activities, camaraderie, and solidarity across the document and interview material.

The document material frequently represented social participation through collective, profession-based, and family-inclusive activities, including clubs, competitions, excursions, and community events. In contrast, the interview accounts more often described informal and individually initiated activities with close colleagues. This difference may reflect a broader shift towards more individualised forms of social engagement, although it may also partly reflect differences between the two data sources and what they capture. The change may reflect broader societal transformations, such as the increasing centrality of individualized forms of social engagements [28] or digital forms of social connection that reduce reliance on shared activities [29]. It might also reflect occupational transformations, such as more complicated life demands [30].

While the document data presented social engagement initiatives that included families and, in many cases, extended to the broader community, families could feel excluded from the togetherness experienced by officers. This may be a potential downside of a strong sense of belonging within the police force. The document material contained both examples of family-inclusive social activities and accounts of spouses feeling excluded from work-related discussions. This juxtaposition suggests that strong occupational cohesion may coexist with tensions in family inclusion, particularly when work-related experiences are difficult to share outside the occupational group. However, the material does not allow conclusions about how common such experiences were, nor whether family-oriented social activities became less frequent over time. Similar relational tensions have been described in previous research on policing, where family members have reported limited access to officers’ work-related emotional experiences [31,32]. At the same time, camaraderie in the present study was associated with solidarity and belonging among officers, suggesting that occupational social participation may constitute an important source of social support in high-stress professions.

Across the document and interview material, social activities were associated with social connection, mutual support, and opportunities for emotional support. This is consistent with Wilcock’s [11] view of belonging as an integral dimension of health through social connection to others. In policing, such social connections may be particularly relevant given the psychological demands associated with police work, and social support is well established as a protective factor following potentially traumatic events [15]. In the present study, camaraderie and trust were recurrently represented and experienced within professional socialisation, including during police education and through everyday interaction with colleagues. This aligns with previous research describing how shared occupational experiences contribute to trust and cohesion among police officers [33]. Shared experiences, teamwork, and responsibility may further reinforce solidarity in a high-stress occupational context [5]. The high membership rate among officers in the Swedish Police Union may also reflect this sense of community and commitment to mutual support [34].

Our findings also contribute to understanding how social participation is associated with solidarity and belonging within policing, a profession characterised by a strong internal culture and state-sanctioned authority [5]. The findings align with Wilcock [5] and Hammell [35] in highlighting the importance of social connectedness and belonging within meaningful participation. Across the document and interview material, shared activities such as training, music, recreation, and other social initiatives were recurrently associated with collegial relationships, camaraderie, and belonging. This suggests that “doing together” may contribute to a sense of connection within the occupational group. Filstad [36] similarly describes how officers’ motivation to contribute and make a positive impact may foster pride and identification with colleagues. Hammell [35] further emphasises that meaningful social activities can support belonging and well-being. In the present study, belonging emerged as a recurring dimension of social participation, including in accounts of social connections that continued beyond active employment. As belonging also emerged as a central aspect of social participation for the officers in the study, our findings indicate that shared social activities might play a role in fostering camaraderie at work, where ‘doing together’ fosters a sense of long-lasting belonging, which in turn reinforces solidarity. One example was the continuity of social bonds after retirement, which underscores how camaraderie can foster long-term social engagement and even improve officers’ well-being. This is supported by a review of social relationships and health, which reveals that social connectedness is linked to the cognitive functioning of healthy older adults [37].

While solidarity among officers was evident in our data through accounts of loyalty, support, and commitment to the occupational group, previous research suggests that strong identification with the professional role may also blur work-life boundaries [6,32]. In this sense, “living for the job” may function both as a source of meaning and as a potential challenge, as work-life imbalance among police officers has been associated with poorer mental health [38]. According to Granér [5], police solidarity, shaped by shared experiences and work pressures, strengthens group identity and fosters an “us-versus-them” mentality. While it supports well-being and norm stability, it may also distance patrol officers from, for instance, leadership [5]. In the present study, solidarity was represented through practices such as supporting colleagues during health crises, responding to trauma, advocating for workers’ interests, and providing peer support and education. These findings illustrate the dual nature of occupational solidarity: it may support belonging and mutual trust within the occupational group, while previous research suggests that strong internal boundaries may also affect relationships beyond the group [39]. While our findings don’t have community implications, the officers’ social activities appeared to foster participation and solidarity through community engagement. We also focused only on officers, but the results may point to the value of broader social engagement. For example, research on community policing [40] suggests that community engagement supports non-coercive methods and helps reduce social distance, thereby enhancing public trust [40]. These types of broader social engagements should be studied in future studies.

Our findings also illustrate that solidarity and group cohesion coexist with tensions around inclusion and difference. Gender-related challenges, discrimination, bullying, and sexual harassment were represented in the document material, while the interviews provided mixed contemporary accounts of gender dynamics within close teams. Previous research suggests that strong internal solidarity may shape group norms and perceptions of difference and, in some contexts, contribute to exclusionary behaviours [41]. These dynamics highlight the potential duality of occupational solidarity: it can support cohesion and belonging while also reinforcing established norms that may make inclusion more difficult when group identities are challenged.

Despite progress, barriers remain, including stigma around mental health, and previous research links sexism in male-dominated workplaces to reduced belonging, poorer mental health, and lower job satisfaction [42]. Our findings indicate both resilience and challenges in how officers address mental health. Solidarity appears to support resilience through mutual support, making help-seeking a vital process. Nevertheless, according to previous research, stigma remains a barrier as officers often avoid individual assistance to uphold an image of strength and self-reliance [43]. This duality suggests that while camaraderie can promote resilience, it may also discourage vulnerability and hinder help-seeking, underscoring the need for further research on how to balance these dynamics. The duality highlights the complexity of occupational solidarity: the same social bonds that enable emotional support and resilience may simultaneously reinforce norms of toughness and self-reliance that discourage formal help-seeking. For instance, recurring references to alcohol across decades, even if not mentioned in the interviews, highlight how substance use is framed as an occupational and social concern within policing, and has been for a long time. The document data addresses the topic through preventive and educational initiatives. According to international research, substance use is considered a risk factor for police officers [44,45]. Its recurrent discussion within the Swedish police over decades may be interpreted as indicative of informal coping, in which psychological strain is self-medicated by work characteristics rather than through formal help-seeking behaviour, also supported by prior research on police officers’ alcohol use [44,45].

Levasseur et al.’s taxonomy [3] was chosen for its relevance in distinguishing levels of social participation, particularly within health research. However, our context, a high-demand, male-dominated profession with a strong internal culture, differs from prior applications focused on ageing and health. This required a critical examination of the taxonomy’s applicability. During the deductive phase, we identified patterns, especially collective, emotionally charged activities that fostered solidarity and belonging, which did not fully align with the existing framework. This prompted an inductive phase to extend and contextualise the taxonomy for other settings. By fostering environments ranging from informal gatherings to formal collaborations, these initiatives appeared to support social participation and reinforce purpose and connection, though expressions and priorities varied historically. In addition, our findings suggest extending Levasseur et al.’s taxonomy [3] to encompass expanded formal duties, offering a safety net for officers facing the profession’s physical and emotional challenges and highlighting the importance of solidarity. These findings align with recent research proposing a new taxonomy of social participation, which describes a category encompassing participation in relaxation and support groups as activities that foster a sense of group belonging [46]. Similarly, in line with Barsade and O’Neill [47], belonging shaped by group norms may reduce emotional exhaustion, which Wilcock [11] also supports, as both doing and belonging underpin well-being.

### Limitations

Due to differences in data type and historical scope, changes in social participation should be interpreted with caution, focusing on how participation was experienced, narrated, and valued within the profession. The following limitations should also be taken into account: The document data, drawn from police union journals, may reflect a selective perspective, as they exclude employer views, and data on specific events declined over time, possibly due to shifts in journal focus or cultural changes. However, the broad timeframe (1904–2023) allows analysis of long-term trends, with interviews linking past and present social participation and solidarity from a personal perspective of the police officers.

This study’s focus on the Swedish police limits transferability to other groups or contexts. However, it provides a foundation for comparative research across national and professional cultures. For instance, occupational cultures vary across both national and professional contexts. While Swedish policing is characterised by a strong welfare-state orientation and high union membership, solidarity and social participation may be structured differently in more hierarchical or adversarial systems. Furthermore, although women were included, the limited number of female participants restricts broader conclusions about gender dynamics across the police force. Future studies should clarify which aspects of solidarity and participation are context-specific and which are generalisable.

For clinicians, the findings highlight the value of supporting meaningful social activities tailored to the cultural and structural conditions of occupational groups, especially where stigma and masculine norms may hinder help-seeking. However, the absence of longitudinal data limits causal inferences about changes in social participation over time. Interview responses may have been shaped by social desirability, given the group-oriented police culture and sensitivity around mental health, cohesion, and exclusion. These factors, along with varying data sources and historical contexts, warrant caution when interpreting changes in participation over time.

## Conclusions

Social activities may have historically supported strong bonds and a sense of belonging among police officers and continue to do so. The findings extend theoretical understanding of social participation and solidarity, offering guidance for designing activity-centred and inclusive social environments in high-stress professions. Methodologically, illustrates how integrating document and interview qualitative data opens an enriched perspective on the evolution of occupational practices, providing a methodological pathway for examining continuity and change across other professional contexts. Furthermore, the study highlights how occupational cultures may shape the types of activities, meanings, and health implications of social participation over time. Understanding these dynamics may inform organisational strategies to strengthen cohesion and well-being in other high-demand professions besides the police context. For clinicians, the findings underscore the importance of supporting culturally aligned social participation, especially where stigma limits help-seeking. For instance, occupational therapists or psychologists can draw on historical patterns to understand how professions sustain or resist health-promoting practices over time.

## Supporting information

S1 File Supplementary file. (DOCX)
