# Supplementary material for: A qualitative study of police officers’ experiences of solidarity and belonging through social activities
Source: PLoS One. 2026 Nov 12;21(11):e0360545. doi: 10.1371/journal.pone.0360545 (PMC13759074; doi:10.1371/journal.pone.0360545)
Supplement: Supplementary file 1 [file pone.0360545.s001.docx]

Supplementary file. Historical Data from Police Union Journals, Searches Between 1903 and 2023.

| **Decade** | **English Title** | **Description** |
| --- | --- | --- |
| 1904–1913 | Occupy Yourself! | Encouragement to prioritize education over leisure. |
|  | The Idea of the Summer Retreat | A proposal to create a retreat (rest home) for police is discussed at the annual meeting. |
|  | Joy of Work | The importance of encouragement from officers in a thankless job is emphasized. |
|  | Team Spirit | Police are encouraged to promote camaraderie through unity and solidarity. |
|  | Unified Cooperation! | The Police Union urges all police officers to join the union. |
|  | Gothenborg Police Choir | A choir has been formed in Gothenburg, with a description of the activities and members. |
|  | Training for Leadership | Emphasis on recruiting capable and suitable officers. |
|  | Excessive Humanity Can Lead to Neglect of Duty | Officers should have humanity but not let it completely guide their actions. |
| 1914–1923 | Police Recreation Home | Revived proposal for a recreation home for police, financed by a lottery. |
|  | Cultivation Leave in Stockholm | City employees get two days of gardening leave; police officers get one day with a rest day. |
|  | Lottery for the Police Officers' Rest Home | A lottery was approved to finance a recreation home for police officers. |
|  | Chess | Chess results from Stockholm |
|  | To the Association “Kamraterna” | The new chairman of Kamraterna discusses future challenges. |
|  | How Should the Weary Best Spend Their Vacation | A doctor advises on optimal recovery during vacation. |
|  | Once Again on Swimming | Stockholm police officers are encouraged to participate in swimming lessons ahead of competitions. |
|  | Ahead of the Scandinavian Police Sports Competition | Police officers from three Nordic countries will compete in Copenhagen, with a focus on Swedish traditions. |
|  | After the Sports Battle | Swedish police officers won overwhelmingly at the Nordic Games in Copenhagen. |
|  | Both Leaders and Comrades | Constables and officers should treat each other as colleagues without making a friendly distinction. |
|  | Mutual Understanding | Emphasizes the importance of unity within the police force for better working conditions. |
|  | Open Forum | Opposition within Kamraterna against those causing division is discussed. |
|  | Corps Spirit | Description of historical corps spirit and the risks of excessive internal isolation. |
|  | Cooperation | Cooperation between constables and officers is poor, likely due to fear. |
|  | Team Spirit, Mutual Understanding, and Tolerance | Emphasizes the importance of cooperation and understanding within the Kamraterna union. |
|  | Unity Within Our Ranks Is Our Strength | A call for unity and brotherhood within the police force in connection with the union's 20th anniversary. |
|  | Stockholmers on Outing | The Kamraterna union organized an outing to Djursholm with music. |
| 1924–1933 | Social Reflections on the Alcohol Issue | Reflections on alcohol's impact on social conditions. |
|  | Experiences as District Sheriff During the Ådalen General Strike 1909 | A lecture on experiences during the 1909 Ådalen general strike was given at a city attorneys' meeting in Stockholm. |
|  | The Stockholm Police Christmas Party | Christmas party at Berns Salonger organized by the police themselves, with some criticism of higher officials. |
|  | At a Party with the Stockholm Policemen | A three-day party in Stockholm, with the first day dedicated to children, included singing and performances. |
|  | Comrades' Party in Djursholm | A description of the supper and festivities at a party in Djursholm. |
|  | Evening Entertainment for Helsingborg Police and Their Families | The Helsingborg police study circle organized an event with music and a speaker who was a former sailor in Antwerp. |
|  | Stockholm Police Choir's Children's and Family Party | A children's and family party at the Grand Hotel on January 7th with dancing, gift distribution, etc., costs 2 SEK to attend. |
|  | Stockholm Police Annual Party | An annual party at the Grand Hotel in February costs 2 SEK, with performances by the choir and a jazz orchestra. |
|  | Gothenburg Police Officers' Chess Club | Announcement from the tournament committee regarding the autumn tournament, including dates. |
|  | Open Forum - Vacation and More in Södertälje | Argumentative text about vacation planning |
|  | Police Officers' Sporting Interests | Sports are recommended as important for physical and mental health within the force. |
|  | Sundsvall Police Sports Association | Information about Sundsvall police's annual meeting and sports results, including wrestling. |
|  | Response to “Lund Interiors” | A previous comment on the Lund police commissioner is revisited, with a differing opinion. |
|  | Corps and Comradeship Spirit | Discussions about corps spirit and the problems within the union. |
|  | Comradeship | The editorial calls for an end to the private discussion on comradeship within the Lund Police. |
|  | Comradeship | Criticism of poor comradeship within the Lund police force, with suggestions for improvement. |
|  | Comradeship | An overview of the terms "comrade" and "comradeship" along with a related discussion. |
|  | "We Should Take Care of Each Other" | A text on collegiality urging against division within the union. |
|  | Collective Punishment | A police commissioner in Lund is criticized for using collective punishment, which is legally prohibited. |
|  | Bullying | A short story about an officer's abuse of power on Christmas Eve at Gävle police station. |
|  | Stockholm Police Choir Celebrates 10th Anniversary | A Christmas party celebrated the choir's founding in 1918 and its activities in 1929 with singing and supper. |
|  | The Police Union's Planned Vacation Trip | A trip to Paris and Switzerland, combining vacation and study, is planned for July 5-18. |
|  | Fourth District Shooting Section | The annual meeting reported on shooting competitions and other activities. |
|  | Malmö Police Pistol Club | Information about results from pistol tournament. |
|  | Family Outing | A March outing organized by the Stockholm Police Sports Association included skiing and a shooting competition. |
|  | Uppsala Police on Pleasure Trip | Uppsala police went on an outing to Västerås on June 7th, with visits to the cathedral and ASEA's workshops, followed by coffee. |
|  | Entertainment | A city council report on inspections of unmarried police constables' dormitories was considered entertaining. |
| 1934–1943 | Interaction and Environment | A review of a book leads into a professor's remarks on positive social interaction in folk high schools and the dangers of alcohol. |
|  | Singers' Festival at Fyris | A concert by the combined male choirs of the police and fire brigade with music and performances for 80 people, ending at 2 AM. |
|  | Police Christmas Party in Norrköping | A Christmas party in Norrköping with 100 guests, including 35 children, with a simple supper and dancing until after midnight. |
|  | Midwinter Celebration in Vänersborg | A midwinter festival with around 40 people gathering. |
|  | "The Brotherhood" Celebrates Christmas in Lund | A Christmas party in Lund with supper and socializing, ending at 2 AM. |
|  | Christmas Party in Kalmar | A Christmas party in Kalmar for entire families, emphasizing the sense of unity from the highest to the youngest constable. |
|  | Don't Forget - Stockholm Police’s Traditional Children and Family Celebration | An invitation to the traditional children's and family party in Stockholm. |
|  | Police Party in Uppsala | A Christmas party at the Stadshotellet in Uppsala, starting with the children and continuing with the adults until 2 AM. |
|  | Thoughts on the Christmas Season | Reflections on the meaning of Christmas, emphasizing stillness and self-reflection. |
|  | A True Sense of Camaraderie | A description of the importance of comradeship in collective contexts, emphasizing the need for unity within the union. |
|  | Unification of Police Organizations | A call for the unification of the fragmented police unions in cities and rural areas. |
|  | Police and Fire Brigade Concert in Helsingborg | A concert in the town hall's assembly hall in March with the police and fire chief present. |
|  | Danish Police Choir Visits Stockholm | The Danish police choir visited Stockholm, performed at Skansen, and toured the city. |
|  | Sweden's Oldest Police Choir Turns 35 | About Gothenburg's police choir celebrating its 35th anniversary, consisting of 33 members. |
|  | Lund Police Officers' Shooting Club | Several practice and competition shooting events have been held. |
|  | Karlskrona Police on a Summer Outing | A summer outing for members to the Blekinge archipelago, including swimming, with children in attendance. |
| 1944–1953 | The Sobriety Association Tre Kronor | The sobriety association Tre Kronor held a meeting with various activities, including an outing and Christmas party, and discussed the dangers of alcohol. |
|  | Stockholm's Auxiliary Constables Form a Discussion Club | A meeting at Bergsgatan 23 to discuss working conditions, leading to a discussion about forming a discussion club. |
|  | Collecting Uniform Badges – Also a Hobby | A police officer describes his hobby of collecting uniform emblems such as buttons. |
|  | Chess Enthusiasts! | A proposal from Östersund to form a national chess club, with correspondence games and tournaments. |
|  | Police Chess Federation | A Stockholm police officer agrees with the idea of establishing a chess club proposed by the Östersund police. |
|  | Personal Activity and Cooperation | A description of the benefits of interacting with others for personal development. |
|  | Police Sports in Gothenburg | Descriptions of skiing, orienteering, wrestling, and weightlifting in Gothenburg. |
|  | Swedish Police Sports Federation Turns 20 | A review of the activities of the Swedish Police Sports Federation over the past 20 years. |
|  | Orienteering | Descriptions of an orienteering competition involving orienteering, shooting, and grenade throwing with participants from the police, fire brigade, customs, and tramway workers. |
|  | Invitation | Sundsvall police invite others to a 30 km cross-country race, a 3x10 km relay, and pistol shooting on skis. |
|  | Winter Chronicle from Gothenburg | Two junior championships in weightlifting were won by the Gothenburg Police Sports Association. |
|  | Malmö Police Officers Head to Paris | Five police officers from Malmö will compete in a fencing competition in Paris, with plans to visit various police departments while there. |
|  | Ahead of the Orienteering Championship in Östersund | Information about an upcoming competition (Swedish Championships) in orienteering in Östersund. |
|  | Dalarna's District Orienteering Championship | District Championships in Dalarna in orienteering. |
|  | Malmö Police Officer Wins Swedish Championship in Saber Fencing | A police officer from Malmö has become the Swedish champion in saber fencing and will compete in Paris. |
|  | Stockholm Police Track and Field Athletes | A description of Stockholm police's activities in track and field. |
|  | Invitation | An invitation to competitions in track and field, shooting, pentathlon, swimming, and more in Borås. |
|  | The Bobbies - The Singing Police Officers | Five constables with a passion for singing, supported by colleagues who swapped shifts, have performed in folk parks. |
|  | Malmö Police Christmas Concert | A Christmas concert in the main hall of the Malmö police station, with several high-ranking officers in attendance. |
|  | Stockholm Police Band | The Stockholm Police Band held its annual meeting and needs to recruit new members. |
|  | Police Service | The first Swedish police church service was held in Kungsholms Church in April 1945, with a full church and the Stockholm Police Choir performing. |
|  | Winter Trips to the Mountains | Hundreds of winter destinations are recommended by the Tourist Association for those taking winter vacations. |
|  | With TCO and TBV to Scotland, Holland, and Italy | Information about leisure trips organized to Scotland, Holland, and Italy. |
|  | Sport Shooting in Stockholm Begins | Shooting in the basement of the police station, using a Walther pistol, with competitions held on Thursday evenings. |
|  | Shooting and Ball Talk from Gothenburg | A description of shooting with various calibers and a discussion about handball. |
|  | Shooting Highlights from Örebro | A description of the shooting sports linked to the Örebro police force. |
|  | Pistol Shooting in Helsingborg | A review of the past year's activities for the Helsingborg police shooting club. |
|  | Uddevalla Police Pistol Shooting Club | The annual meeting was held, with a review of the past year's activities. |
|  | Karlskrona Police Sports and Shooting Association | Description of Karlskrona Police's sports and shooting association. |
|  | Police Academy Social Gathering | A convivial gathering at Berns with teachers and students, where students were encouraged to confide in teachers if they had problems. |
| 1954–1963 | The Swedish Policemen's Total Sobriety Association | A description of the Swedish Police's sobriety association and information about the social dangers of alcohol, youth crime, and more. |
|  | Study Work | A pastor held a study circle in philosophy for 10 police officers from Västerås. |
|  | Group Trip to Italy | Information and invitation to a group trip to Italy, Switzerland, and Austria. |
| 1964–1973 | Four Comrades | A discussion about the importance of camaraderie within the union, emphasizing mutual support among police officers. |
|  | "With Song in Our Hearts…" | Description of Motala Police Choir, which has 20 members practicing once a week. |
|  | What to Do When Retired? | Encouragement to participate in study circles after retirement. |
|  | Police Bridge Championship 1971 | The Police Swedish Championship in Bridge was held in Stockholm with 46 pairs from 25 police districts. |
| 1974–1983 | Happy Community or Escapism? | The Swedish Police Sobriety Association writes about the dangers of alcohol. |
|  | Benefit with Pleasure in Västerås: Skipper Course and Bug Dance | Several courses were conducted in Västerås during the year, including dance and skipper's certificate courses. |
|  | Stockholm Criminal Association Turns 70 | The Stockholm Criminal Police Association celebrated its 70th anniversary. |
|  | "Health Course for Police Officers and Railway Workers at Storvallen" | The Swedish Police Sobriety Association invites police officers and railway workers to a wellness course in the mountains, with daily morning exercises and hiking. |
|  | Gothenburg Police Stamp Club Turns 10 | 45 stamp-collecting police officers in Gothenburg celebrated the club's 10th anniversary. |
|  | Physical, Mental, and Social Issues When Working Unusual Hours | A research report highlights the physical, psychological, and social problems associated with working irregular hours. |
|  | Working Together Means Willingness – Not Just Ability | A female police officer sent in a letter discussing the working conditions for women in a male-dominated police world. |
|  | How Does a Police Family Experience Public Interaction? | An article series about three police officers from Östermalm district and their wives discussing the officers' work situation. |
| 1984–1993 | Alcohol-Work-Life-Peer Support at the SPHF Conference in Spånhult | The Swedish Policemen's Temperance Association held a conference in Småland discussing alcohol, work life, and camaraderie. |
|  | JHF Course Open for SPHF Members | The Railway Employees' Temperance Association and the Swedish Teachers' Temperance Association invited members to a course in the mountains, open to the Swedish Policemen's Temperance Association with 15 spots. |
|  | Course Five | Study circles that go beyond police topics, such as philosophy, are organized, including for pensioners, with study trips also arranged. |
|  | Interesting Police Research Symposium: Police are More Stress-Resistant Than Others | A police research symposium was held in Stockholm, presenting findings that police are more stress-resistant than other professionals. |
|  | The Oldest Active Study Circle is in Gothenburg? | A study circle in Gothenburg, active since 1956, has had about 17,000 participants over the years, covering topics like economics, leadership, philosophy, and hobbies. |
|  | The Country's Oldest Police Association Celebrates its 90th Anniversary | 165 guests attended Landskrona Police Association's 90th-anniversary celebration. |
|  | Police Day in Vällingby | The police in Vällingby celebrated their 25th anniversary with various demonstrations for the public. |
|  | Organize Wellness Weeks in Snasalien for Police Officers | Misuse problems among police have significantly increased in recent years due to a stressful work environment, media pressure, and issues with supervisors and colleagues, leading to a proposal for wellness weeks at Snasalien in the mountains. |
|  | Stress Issues and the Psychosocial Work Environment Are Addressed Specifically | The police ordered health checks from Statshälsan to address stress and psychosocial work environments. |
|  | Dog Championship in Mora | The Swedish police K9 championship was held in Mora. |
|  | Challenges for Women: "Prejudices Confirmed in Statistics" | It's harder for female police officers to be promoted compared to their male counterparts. |
|  | Policewomen Recognize Each Other’s Challenges | A Swedish female police officer spent time in England with policewomen from across Europe. |
|  | "The Wife as Important Psychological Support" | Among other police officers, true friendships exist, making spouses feel left out when their husbands talk about work. |
|  | Friendships Last Since SPF’s First International Trip in '51 | In 1951, a study trip to Germany led to a long-lasting friendship between a Swedish and a German police officer. |
|  | You Can Certainly Trust a Jehovah’s Witness! | A letter to the editor questions whether a Jehovah's Witness can be trusted as a police colleague, concluding that they can and have a place in the public sector. |
|  | I’m Gay, a Communist – and a Police Officer | A retired police officer talks about his career and identifies as homosexual, communist, and a sober alcoholic. |
|  | The Pros and Cons of the Police Profession | A survey has been conducted about the impact of police work on family life. |
|  | Sexual Harassment in the Workplace | The National Working Environment Board is taking sexual harassment at workplaces seriously. |
|  | My Male Colleagues Broke Me – I’ll Never Go Back to Them | An anonymous female police officer shares how male colleagues broke her down psychologically with bullying and sexual harassment. |
|  | Mariapolis Police Gathering | A reunion was held for former police officers from the Maria district. |
|  | Stockholm Police Retirees Have Many Activities Underway | Retired police officers in Stockholm have various activity groups like walking, sauna, and museum groups. They also manage a fund for the widows and children of fallen officers. |
|  | Veteran Club in Kalmar | A pensioners' association has been formed in Kalmar with 18 members. One of the first activities was an excursion to "Glasriket" and a visit to Nybro police station for coffee. |
|  | A Dedicated Veteran Association for Vimmerby Police | A veteran association has been formed in Vimmerby to allow retired police officers to meet regularly and maintain camaraderie. |
|  | Police Retirees’ Association in Malmö Celebrates 50 Years | One of the oldest police pensioner associations celebrated its anniversary with about 150 participants, including women. |
|  | The Västerås Police Retirees' Association Celebrates 35 Years | The Västerås police pensioner association celebrated an anniversary with the sentiment that "Once a police officer, always a police officer." |
|  | High Activity in Umeå Police Retirees' Club | The Umeå police pensioner association held its annual meeting. |
|  | Extensive Activities Among Gothenburg Police Retirees | Gothenburg's police pensioners run activities with meetings, study visits, and more, and have 268 members. |
|  | Police Veterans Form a Club for Jönköping County | A veterans' club for police pensioners has been formed in Jönköping. |
|  | New Chair and Secretary Elected for Nyköping Retirees | The Nyköping police pensioners' association held its 11th annual meeting. |
|  | Sundsvall Police Veterans Appoint New Chair | Sundsvall's police veterans held their annual meeting and elected a new chairman. |
|  | Veterans Club Also in Mora | A pensioners' association has been formed in Mora. |
|  | Kristianstad Now Has a Senior Club Too | A pensioners' association has been formed in Kristianstad. |
|  | Veterans with Spirit | The Karlskoga police veterans' association visited the Bofors weapons industry. |
|  | Karlstad Veterans on a Bus Tour | Karlstad veterans went on a bus tour around the county, including a visit to a power station. |
|  | Aid Fund for Elderly Police Officers in Stockholm | Stockholm police pensioners manage two support funds to help retired police officers in financial difficulty. |
|  | All-Christian Police Conference in Gothenburg, September 18-20 | Christian personnel from various police departments meet. |
|  | All-Christian Police Conference in Rättvik, September 22-23 | Invitation to an all-Christian police conference in Dalarna. |
|  | Clay Pigeon Shooters to Italy! | Invitation to participate in a clay pigeon shooting competition in Italy and participants pay for the trip themselves, but accommodation is covered. |
|  | Police Bridge Championship 1984 | The Police Swedish Championship in Bridge was held in Norrtälje. |
|  | Police Chess Championship | Invitation to participate in the police chess championship at Kronoberg in Stockholm. |
|  | Police Bridge Championship 1984 | The police bridge championship is held in Skövde, with police officers, prosecutors, and pensioners invited. |
|  | Police Bridge Championship | The police bridge championship is held in Malmö with police and prosecutors invited. |
| 1994–2003 | Tough Officers Use Gentle Methods | The police special forces focus on selection tests that emphasize self-awareness and maturity. |
|  | The Boss Can Miss It | The police chief in Falun is self-critical regarding the psychosocial care provided after officers shot the murderer Mattias Flink. |
|  | When He's in Full View, I Shoot | A story on the reactions and care after police officers neutralized the murderer Mattias Flink in Falun in 1994. |
|  | Report from a Girls' Talk | A text on the difficulties women face in a male-dominated profession. |
|  | Blunt, Strong, Short Temper | A story on the first female chair of the TCO trade union. |
|  | Policewomen Wrote a Play | A play about female police officers, written by policewomen. |
|  | Deep Sorrow in Stockholm – "Nothing Will Ever Be the Same After the Estonia Disaster" | During the Estonia ferry disaster, 63 Stockholm police employees lost their lives. A memorial was held, with remaining colleagues supporting each other emotionally. |
|  | What Is the Union's Guiding Principle? | A young Stockholm police officer believes the union should view its members as customers rather than something taken for granted. |
|  | Officers Said No – to an HIV-Infected Colleague | An HIV-positive police officer shares how colleagues have distanced themselves from her. |
|  | Unwanted Sex at Work | An article about sexual harassment at work and the line between criminal and non-criminal behaviour. |
|  | Harassed and Discriminated | 15% of female police officers were sexually harassed at work last year, and 41% feel discriminated against because of their gender. |
|  | Have You Encountered Racism Within the Force? | A retired police officer responds to the question of whether racism exists in the police force, expressing surprise given recent ethics training. |
|  | Reassigned Without Clear Reasons | About a dog handler who was reassigned to a different police role without clear reasons. |
| 2004–2013 | When the Colleague Drinks | On the increasing alcohol consumption among Swedes and the responsibilities of managers in certain aspects. |
|  | Further to the Boss | Remote management is becoming more common within the police force, but it poses challenges, particularly in maintaining social contact with employees who are rarely seen. |
|  | The Rainbow Festival Attracted Gay Police Officers | Gay police officers participated in a Pride parade in Malmö, with hopes of encouraging more to join in the future. |
|  | Crisis Management: Support in Grief and Despair After the Murder | A police officer was shot dead, and the article discusses how colleagues supported each other afterward and the effectiveness of the crisis support provided by the employer. |
|  | Difficult to Work After a Colleague's Murder | A police officer was shot dead on duty, and colleagues have found it hard to return to work afterward. |
|  | If a Colleague Falls into Crisis | When a colleague is in crisis, it can be difficult to know when to step in and who holds the responsibility. |
|  | Silence is Silver, Speaking is Gold… | Casual conversations at work play a crucial role in creating a positive work environment, improving colleague relationships, and more. |
|  | Conversations on Leadership | An article about leadership in the Umeå police department and how they work with cohesion |
|  | Police Officers Fall in Love at Work | Police officers often find partners at work, with an analysis of the pros and cons of dating a colleague. |
|  | Bullied Police Officer Receives Compensation | Managers have been responsible for discriminatory treatment against a police officer, and it is now recognized as a work injury, with compensation awarded. |
|  | Direct | A profile of a female police officer who discusses the challenges of being a woman in the police force. |
| 2014–2023 | "A Pat on the Back, Then Back to Work" | A debriefing group started in Gävle in the mid-1990s, and it’s noted that attitudes toward psychosocial care have changed over the past 20 years. |
|  | "I’m Half of Rickard Now" | After a traumatic incident at work, a police officer suffers from PTSD, which affects his daily life, and he has had to seek help on his own. |
|  | Saved by Colleagues | A 42-year-old police officer had a stroke due to high work pressure, but afterward, he experienced support from colleagues and his boss. |
|  | Few Follows John – Says the Union | The article discusses police officers who leave the profession, some of whom return later due to the camaraderie in the job, which they can't find elsewhere. |
|  | "This is the Worst Situation One Could Face" | A police officer was shot dead, and the article discusses how colleagues supported each other afterward and how well-functioned the crisis support from the authorities was. |
|  | Expert: The Press Conference Violated the Work Environment Act | Two police officers had a private relationship, and when one was suspected of misconduct, the employer started an investigation that ended in a press conference, after which the police chief took his own life. |
|  | No Major Crisis in Leaving the Police Force | A sociology thesis shows that retired police officers stay busy after retirement, often taking on organizational responsibilities like managing housing associations or other clubs. |
|  | Opportunity to Meet (Each Other) | The police shooting championships were held in Falun, focusing on the competition and the sense of community among participants. |
|  | In Control of the Situation | The Swedish police championship in orienteering is organized in Varberg |
